# Theranostic aspects of palladium‐based bimetallic nanoparticles in biomedical field: A state‐of‐the‐art

**DOI:** 10.1002/hcs2.96

**Published:** 2024-06-11

**Authors:** Shwetha B. Nagarajan, Sanjeevi Ramakrishnan, Anuradha Jayaraman

**Affiliations:** ^1^ NIMS Institute of Allied Medical Science and Technology NIMS University Rajasthan Jaipur India

**Keywords:** antibiotic resistance, bi‐metallic, biocompatible, nanomaterials, palladium, regenerative activity

## Abstract

The exploration of newer antibacterial strategies is driven by antibiotic‐resistant microbes that cause serious public health issues. In recent years, nanoscale materials have developed as an alternative method to fight infections. Despite the fact that many nanomaterials have been discovered to be harmful, numerous researchers have shown a keen interest in nanoparticles (NPs) made of noble metals like silver, gold and platinum. To make environmentally safe NPs from plants, green chemistry and nanotechnology have been combined to address the issue of toxicity. The study of bimetallic nanoparticles (BNPs) has increased tremendously in the past 10 years. The production of BNPs mediated by natural extracts is straightforward, low cost and environmentally friendly. Due to their low toxicity, safety and biological stability, noble BNPs with silver, gold, platinum and palladium have the potential to be used in biomedical applications. They have a significant impact on human health and are used in medicine and pharmacy due to their biological characteristics, which include catalytic, antioxidant, antibacterial, antidiabetic, anticancer, hepatoprotective and regenerative activity.

## Graphical abstract

Theranostic potencies of Pd‐based bimetallic NPs.

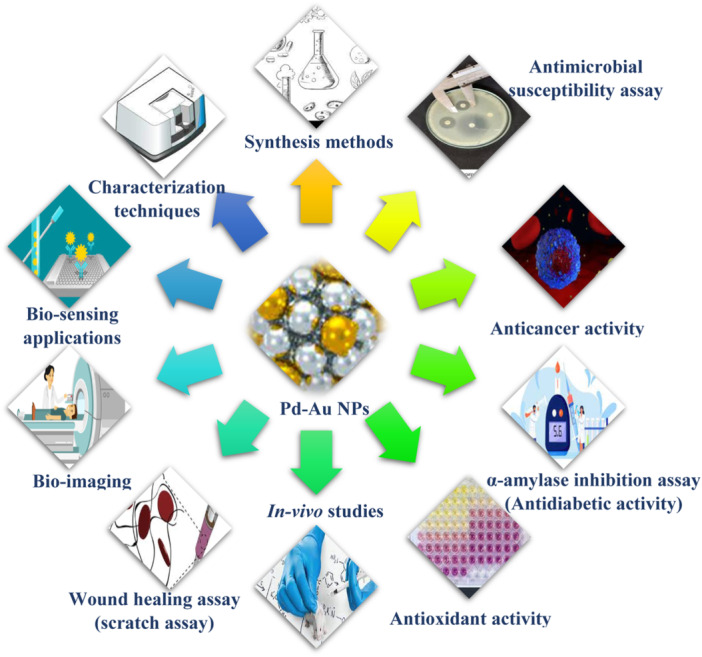

**Abbreviations**
AC: alternating current
Ag: silver (argentum)
Au: gold (aurum)
BNPs: bimetallic nanoparticles
CT: computed tomography
Cu: copper (cuprum)
DC: direct current
DPPH: 2,2‐diphenyl‐1‐picrylhydrazyl
FTIR: Fourier transform infrared spectroscopy
HAT: hydrogen atom transfer
HR‐TEM: high‐resolution transmission electron microscopy
LOD: limit of detection
MNPs: monometallic nanoparticles
MRI: magnetic resonance imaging
NIR: near infrared
NPs: nanoparticles
Pd: palladium
Pt: platinum
SEM: scanning electron microscopy
SPR: surface plasmon resonance
TEM: transmission electron microscopy
TGA: thermal gravimetric analysis
VSM: vibrating sample magnetometer
XPS: X‐ray photoelectron spectroscopy
XRD: X‐ray diffraction

## 1 INTRODUCTION

Nanoscience is a multidisciplinary technology that encompasses physics, chemistry, biology, medicine and material science. Richard Feynman, an American physicist, first proposed the concept of nanotechnology in 1959. The term ‘nanotechnology’ was coined by Norio Taniguchi of Tokyo University of Science in 1974 [1]. The term ‘nano’ denotes one billionth of a metre. As a result, nanoparticles (NPs) are particles with a nanometre scale of 1–100 nm that exhibit properties that are distinct from or superior to the bulk material from which they are created. Catalysis, electronics, biosensors, optical, magnetic and drug delivery systems all use NPs. The characteristics of NPs can be determined not only by their size, shape and content but also by their scientific background and technological uses. NPs have properties ranging from bulk material to atoms and molecules. NPs are classified according to their origin, dimension and structure. According to the classification based on original NPs, they might be natural or artificial. In terms of dimension, NPs can be zero, one, two or three‐dimensional. Depending on their structure, NPs can be liposomes, dendrimers, carbon‐based or metal‐based.

## 2 CONCEPTS OF THERANOSTICS

The combination of the terminologies therapeutics and diagnostics, theranostics signifies the use of therapeutic and diagnostic medications in a single dosage to accomplish both objectives that conventional treatments were unable to achieve separately. John Funkhouser is credited with coining the term ‘theranostics’ in 1998 to refer to a substance that combines therapeutic and diagnostic imaging modalities, but this fundamental idea has been used for more than 50 years to image and treat thyroid disorders. When it came to treating patients with hyperthyroidism and later thyroid cancer, Saul Hertz was the first to do so in 1941. Nanomedicine poses potential for targeted drug delivery, imaging and therapy in the field of theranostics. NOs are able to be engineered to carry therapeutic payloads, imaging agents and targeting ligands, allowing for multimodal theranostic approaches. Drug solubility, stability and controlled release at the disease site can all be improved by these nanocarriers. Furthermore, as contrast agents for imaging, nanomaterials with intrinsic imaging capabilities like gold (Au) NPs or quantum dots can be used [2]. To eventually be able to adjust the therapy and dosage with previously unachievable control, the theranostic field's ultimate goal is to be able to image and monitor the diseased tissue, delivery kinetics and drug efficacy [3]. It is possible to advance the field of nanomedicine towards a time of more efficient and customized treatment methods by individualizing medicine as opposed to taking a ‘one size fits all’ strategy [3, 4]. The field of medicine has undergone a significant revolution with the development of nanotechnology and other pertinent techniques and materials, leading to the emergence of the new field of nanomedicine. Owing to its distinct benefits, nanomedicine is becoming more and more significant in the identification, management and avoidance of illnesses, as a result of the swift advancement of diverse nanomaterials. Promising diagnostic and theranostic tools in nanomedicine, nanomaterials can be functionalized through binding to particular proteins, delivering drugs in particular environments or reaching specific local regions. Biomaterials can now access molecules through biologic barriers with the help of diagnostic or therapeutic agents. This makes it possible for the regulation of molecular interactions and the monitoring of changes in the microenvironment. By modifying their optical, electronic, magnetic and biological properties, nanomaterials can be shaped into a variety of forms, sizes, compositions, surface chemical properties and hollow or solid structures. Such features enable nanomaterials to have major applications in dealing with medical conditions [4]. Theranostic nanomaterials possess qualities that are advantageous as well as disadvantageous in the biomedical field (Table 1).

**Table 1:** Advantages and disadvantages of bimetallic nanoparticles as theranostic agents.

Advantages	Disadvantages
They are biodegradable	Gets collected on own
They are biocompatible	Degradation can be highly toxic
Highly flexible for hydrophobic and hydrophilic carriers	Encapsulation of the drug might be poor
Able to adjust the possibility for surface modification	Scale‐up difficulties
Control precision over the particle properties	Cost of their synthesis is very high
Controlled drug distribution	Involves usage of some toxic chemical elements
Unique drug‐releasing methodologies	Chances of leakage before entering into the cell/tissue

## 3 BIMETALLIC NANOPARTICLES (BNPS)

BNPs, as the name implies, are composed of two distinct metal components. Alloys, core‐shells and contact aggregates are examples of bimetallic nanomaterials. Because of their distinct characteristics, they have piqued the curiosity of the scientific and industrial communities. They outperform their monometallic counterparts when used as catalysts. They are inexpensive, dependable options with high activity and selectivity. As a result, tremendous effort has gone into improving these catalysts. Metal characteristics are governed by the metal combination or type present, how they are mixed, and their size. Because two dissimilar metals are combined, their properties can be modified. For particular applications, the BNP can be designed in a variety of ways. Several methodologies for their synthesis and proper characterization have been established. The most noteworthy of the unique features is the increased electrical properties that arise from bi‐metallization. An electronic phenomenon includes charge transfer and orbital hybridization between component metals. The production of alloys can result in structural changes. The chemical and environmental conditions under which they are synthesized have an impact on their structural properties. The change in the reduction rates of the several metal precursors determines the ultimate structural characteristics of the nanomaterial.

Classification is possible because the properties of BNPs are governed by the size, shape, structure, architecture or order of two different metals. The fundamental categories depend on [5]:

The structure and order of mixing the two metals, that is furthermore categorized as follows:

(1) *Mixed structures*, based on the atom arrangement, material is divided into random and ordered arrangements. A mixed structure with a random organization is an example of an alloyed structure. An intermetallic structure is a mixed structure that has an ordered pattern.
(2) *Segregated structures* are a subcluster structure built of two different metals with a shared interface, whereas a core‐shell structure is made of one metal surrounded by another.

Dimensions of BNPs which are further categorized as follows:

(1) *Zero‐dimensional NPs* are prepared by wet chemical synthesis and the common types are nanospheres (NSs), nanopolyhedrons, nanoframes, and in case of NSs, they are identified as convex and concave NSs.
(2) *One‐dimensional NPs* are nanowires, nanorods and nanotubes.
(3) *Two‐dimensional NPs* are nanosheets, nanoplates and nanoribbons.

**Figure 1:**
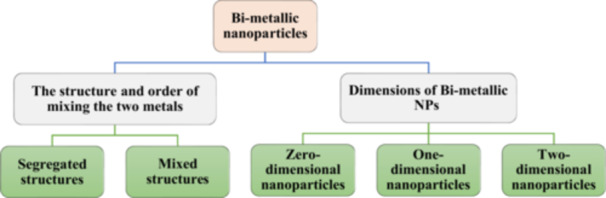
Categorization of bimetallic nanoparticles based on structure and dimensions.

BNPs are formed when one metal combines with another metal in solid form. They can be either substitutional or interstitial structures. A substitutional structure is formed when the sizes of two constituent metals are the same and one metal can substitute for the other in a regular lattice structure. When the size of one metal is less than the size of another, and the smaller metal fits within the atoms of the larger metal, interstitial structures form. The mixing patterns or groupings of atoms of two metals are influenced by bond energy, surface energy, atomic radius, charge transfer and the size of the BNPs. Physical and chemical properties vary according to the number of accessible active sites. The size of BNPs influences their catalytic properties as activity, selectivity and stability. Because zero‐dimensional NPs agglomerate to a larger particle size, reducing surface area and surface energy, they are less stable than dimensional BNPs (Figure 1).

**Figure 2:**
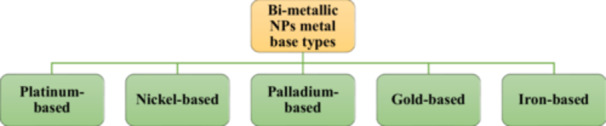
Types of metal bases involved in bimetallic nanoparticles.

Two distinct metals are utilized to generate BNPs. The quantities and mixing patterns of transition and noble metals vary. Based on their constituent metals, BNPs are divided into the following types (Figure 2):

(1) *Platinum (Pt)‐based BNPs*: Pt‐based BNPs, such as Pt(X), where X might be copper (Cu), Au or silver (Ag), exhibit excellent catalytic activity.
(2) *Nickel‐based BNPs*: These are often beneficial due to their catalytic and magnetic properties. These are low‐cost, highly stable and rotate quickly.
(3) *Iron‐based BNPs*: These BNPs have exceptional catalytic activity as compared to iron monometallic nanoparticles (MNPs) or the other constituent metal.
(4) *Palladium (Pd)‐based BNPs*: Pd‐based NPs are used because they are affordable and easy to obtain. Pd‐based BNPs are acid‐resistant and have catalytic and electrochemical properties.
(5) *Au‐based BNPs*: These BNPs are used as biosensors and biomedicines in addition to being catalysts.

The concepts of Pd‐based BNPs are the subject of this review along with their synthesis techniques, characterization techniques, biomedical applications and the disadvantages of Pd‐based BNPs.

## 4 PD‐BASED NPS

Pd‐based NPs are among the most commonly used noble metal nanomaterials. Because of their high specific surface area, abundant active sites and high catalytic activity, Pd‐based NPs have the potential to be efficient catalysts. Publication trends demonstrate the ongoing advancement of Pd‐based NPs and related materials. Pd‐based NPs have valuable catalytic and optical properties that open up a wide range of chemical, medical and environmental applications in human activities. Pd‐based NPs have gained widespread attention in the realm of biological applications due to their significant catalytic and sensing activity. The ability to adjust the size and shape of Pd‐based NPs is critical for facile selective catalytic and sensing characteristics toward various chemical and biological analytes. Pd's relative abundance over other noble metals such as Au and Pt makes it a cheaper alternative for use in different electrochemical sensing and biosensing platforms. Over the last decade, a variety of Pd‐based nanomaterials with variable composition, such as nanocomposites, bimetallic NPs, metal oxide nanomaterials and carbon nanomaterials, have been investigated for the detection of numerous biomarkers due to unique electronic properties, improved catalytic and selective sensing performance. Aside from the typical unique metal characterizations, noble Pd‐based NPs exhibit exceptional physicochemical properties such as high thermal stability, good chemical stability, remarkable photocatalytic activity, electrical properties, optical properties and inexpensive cost. Pd‐based NPs of various sizes and shapes can be made. They can be coated with additional biopolymers or chemicals to produce biocompatible NPs with desired qualities. Previous studies have looked at the wide range of uses for Pd‐based NPs, including organic coupling synthesis, hydrogen storage or sensing, fuel cells, and sensors. Pd‐based NPs, in particular, are widely employed in a variety of applications, including as a catalyst for C–C bond formation and oxidation processes in the pharmaceutical industry. However, lesser studies have been reported on Pd‐based NP uses in the biomedical area. These NPs have recently been discovered to be photothermal agents, photoacoustic agents, gene or medication carriers, and other applications. A few applications on prodrug activation, photothermal therapy and anticancer/antimicrobial therapy were discovered and described at the time in that small review. This review was inspired by the desire to offer current information on the potential applications of Pd‐based NPs in the biomedical field. First, the synthesis methodologies, stabilization procedures and physical properties for the design of Pd‐based NPs for biomedical applications are briefly discussed. Following that, the most recent research on Pd‐based NP applications for cancer/infection PTT (photothermal therapy), photoacoustic imaging, antibacterial application, anticancer therapy, gene and drug transport, prodrug activator, biosensor, and multifunctional NPs is thoroughly discussed [6].

## 5 SYNTHESIS OF PD‐BASED BNPS

**Figure 3:**
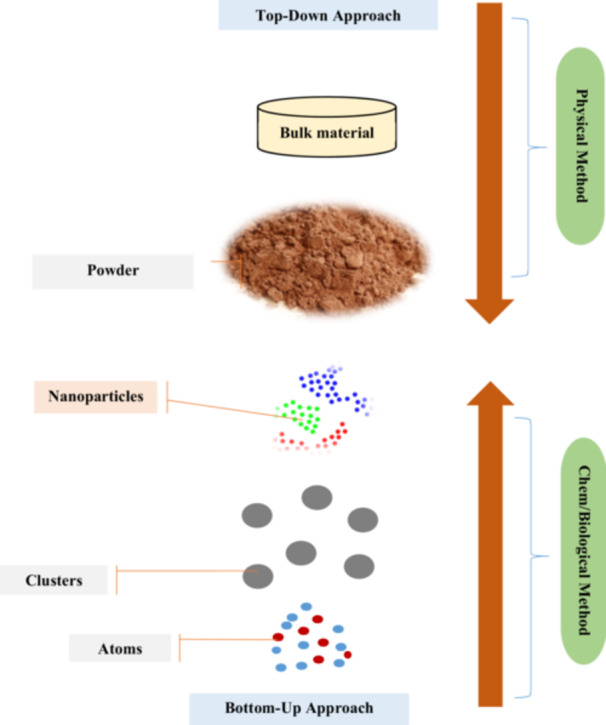
Top‐down approach and bottom‐up approach synthesis mechanisms of bi‐metallic nanoparticles.

NPs are synthesized by two approaches [6, 7] (Figure 3):

i. Top‐down technique that begins with bulk material and progresses through fragmentation by external mechanical forces in the presence or absence of catalysts. Although this process is speedier, there is no control over the shape and size of the BNPs.
ii. Bottom‐up method begins at the atomic and molecular levels and progresses to the nano‐scale level. The size and shape of the object can be changed using this method by adjusting the synthesis settings. This is a slower strategy, but it is far superior to the top‐down approach.

Many factors must be considered when synthesizing Pd‐based nanomaterials:

a. Modification of dimensions and form to maximize the number of chemically active sites.
b. High‐index factor control for improved catalytic activity.
c. To improve stability and activity, bi‐ and tri‐metallic compositions and architectures are being developed.
d. To prepare highly competent catalysts, the essential correlations between the composition, structure and reactivity of Pd nanomaterials must be established.
e. Developing new substrate materials that have high chemical, conductivity and mechanical stability, as well as a large surface area.
f. Allowing for the uniform distribution of Pd‐based catalysts on support materials to improve efficiency.

**Figure 4:**
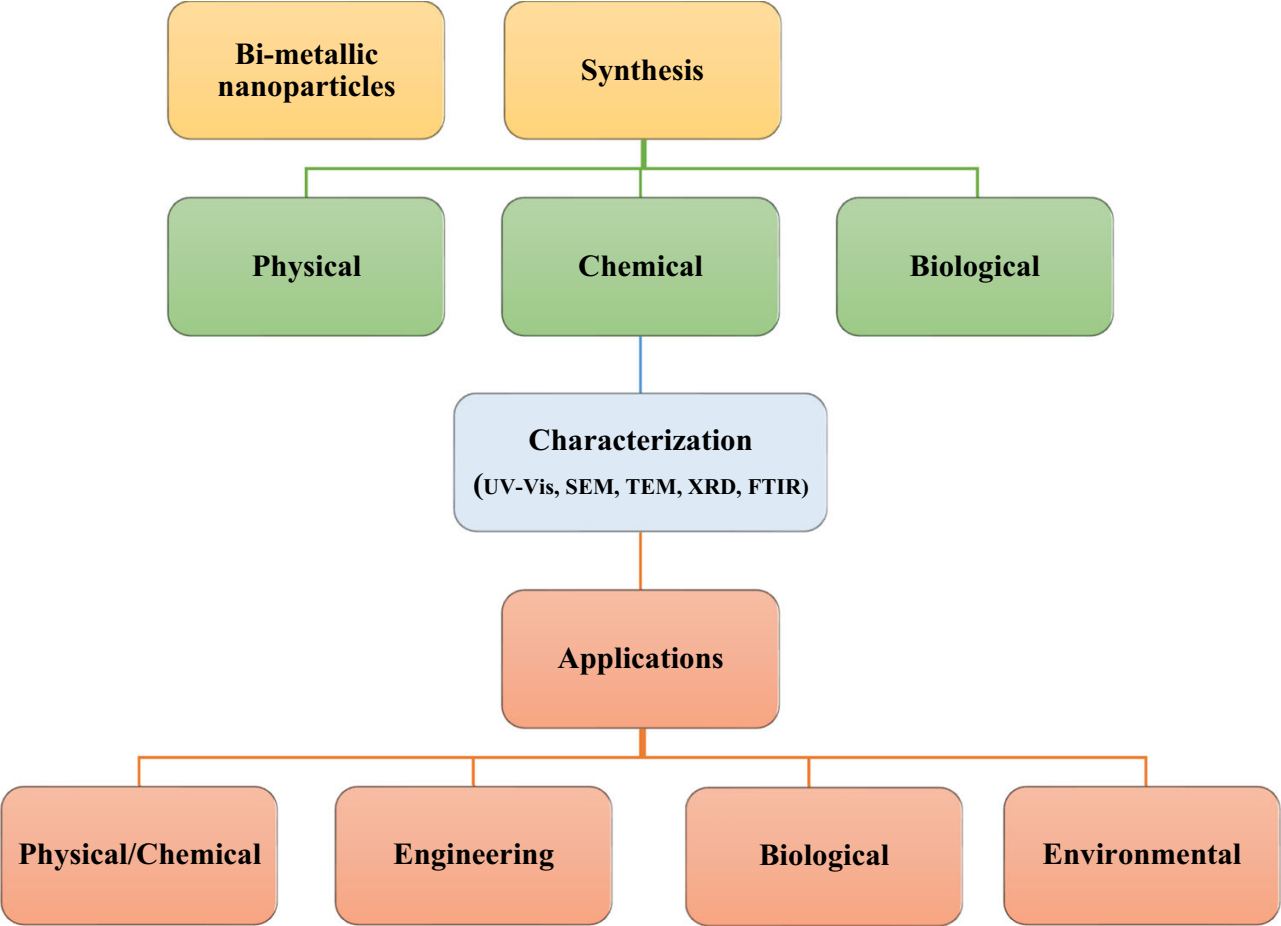
Bimetallic nanoparticles synthesis, characterization and its applications.

Three different methods were used to synthesize Pd‐based bimetallic NPs in this regard (Figure 4), and are described in a tabular format (Table 2):

i. Physical method,
ii. Chemical method and
iii. Biological method.

**Table 2:** The types of BNP synthesis methods along with principles, techniques used, advantages and disadvantages.

Physical method	Chemical method	Biological method
*Principles involved in each method*		
A metal wire is vapourised using a pulsating current and the vapour is subsequently cooled by surrounding gas to create NPs.	The chemical method, on the other hand, was based on the chemical reduction of metal ions to zero‐valent metal atoms and their nucleation to form NPs.	Biogenic nanoparticles (NPs) are produced by biological mechanisms and are more flexible for biomedical and other applications and it is also known as ‘green synthesis’.
*Techniques used in each method*		
Laser ablation, electrical method and microwave irradiation method.	Sol‐gel method, reverse micelle method, sonochemical co‐reduction and radiolytic co‐reduction method.	Parts of plants: leaves, seeds, roots, stem, flower, peels and gums. Secondary metabolites: proteins, polysaccharides and enzymes. Beneficial microbes: prokaryotes (bacteria, archae, etc.) and eukaryotes (yeasts, moulds, algae).
*Advantages in each method*		
No solvent contamination, nanoparticle distribution uniformity, no usage of hazardous chemicals, sterility and size and shape consistency.	Low cost, ease of use, high yield, controlled size and shape, good thermal stability, high uniformity, large‐scale production, variety of particle shapes, and no need for chemical purification.	Provide simple, rapid, potentially more environmentally friendly and cost‐effective methods and can control the size and shape of nanoparticles.
*Disadvantages in each method*		
High temperatures and pressures, as well as costly machinery and energy‐intensive procedures, productivity decrease, nanoparticles' physicochemical properties are altered and inability to regulate particle size.	The use of toxic side products, dangerous solvents and reducing or stabilizing agents.	Size and morphology variations, difficulty in extraction and isolation, production yield are low, seasonal or climatic variations and strong reducing agents are needed.

### 5.1 Physical methods

#### 5.1.1 Laser ablation method

Laser synthesis of BNPs can be accomplished in two ways: top‐down or bottom‐up. Depending on the experimental settings and laser radiation parameters, various nanostructures with chemical, magnetic, optical and electronic properties can be created. When a laser beam strikes materials, the incident light may be absorbed or reflected. There are two sorts of mechanisms involved in this procedure:

i. Photochemical
ii. Photothermal and Photophysical

#### 5.1.2 Electrical method

Electrical approaches for NS synthesis involve the creation of plasma, either in the gas phase or in the liquid phase, using an electric source. As a result, the approaches can be categorized as follows:

i. DC arc discharge method,
ii. AC arc discharge method, and
iii. Pulsed arc discharge method.

#### 5.1.3 Microwave irradiation method

Unlike UV, visible light, and infrared radiation, microwave irradiation promotes energy transfer by direct chemical interaction in electromagnetic fields. Microwave irradiation generates volumetric heating by delivering energy throughout the material's volume. Volumetric heating with microwave energy produces a more uniform and quicker temperature rise than traditional heating methods. Microwave heating is the process of converting electromagnetic energy into thermal energy. Two fundamental concepts govern microwave irradiation:

i. Dipolar polarization mechanism,
ii. Conduction mechanism.

### 5.2 Chemical methods

#### 5.2.1 Reverse micelle method

The polar element aggregates toward the centre, containing a small amount of water in a pocket‐like structure, while the nonpolar tails are exposed to the nonpolar solvent. Micellization of this sort results in the development of reverse micelles or inverted micelles. Surfactants and surface active reagents are critical in the creation of micelles and reverse micelles. Surfactants are classified as follows:

i. Ionic surfactants,
ii. Non‐ionic surfactants.

#### 5.2.2 Sol‐gel method

The bottom‐up sol‐gel approach of NP preparation is used. The sol‐gel process is based on colloidal chemistry, in which a colloidal liquid is chemically converted into a gel state. Starting ingredients for NP synthesis are typically inorganic metal salts or metal‐organic compounds such as metal alkoxides, which are hydrolysed and polymerized into a colloidal suspension or ‘sol’ form. Spin coating or deep coating converts colloidal sol to ‘wet gel’. The chemical change of sol to gel occurs due to particle contact via van der Waals forces or hydrogen bonding. Gelation is the transformation of a sol into a gel.

#### 5.2.3 Radiolytic co‐reduction

The radiolytic coreduction method of preparing BNPs can be carried out under relatively mild reaction conditions such as ambient pressure and room temperature. The approach is very reproducible and effective for producing uniformly sized and highly monodispersed metallic NSs. X‐rays, gamma rays, UV radiation and electron beams are some of the ionization radiations employed in radiolysis. In an aqueous media containing a stabilizer, radiolytic coreduction can be utilized to prepare NSs. The chemical reduction agents are substituted with hydrated electrons with a very high reduction potential in this case. This happens when there is no oxygen present, and the metal precursor is reduced to zero‐valent metal atoms.

#### 5.2.4 Sonochemical co‐reduction

Sonochemistry for NP synthesis incorporates ultrasound into chemical processes. Acoustic cavitation, or the development, growth and implosive collapse of bubbles in liquids bombarded with high‐intensity sound, exhibits the principles of sonochemical reaction. Cavitation causes bubbles to collapse, resulting in extremely high and intense local heating, pressures of 1000 atm and temperatures of 5000 K. This has rapid heating/cooling rates as well as liquid jet streams.

### 5.3 Biological methods

**Figure 5:**
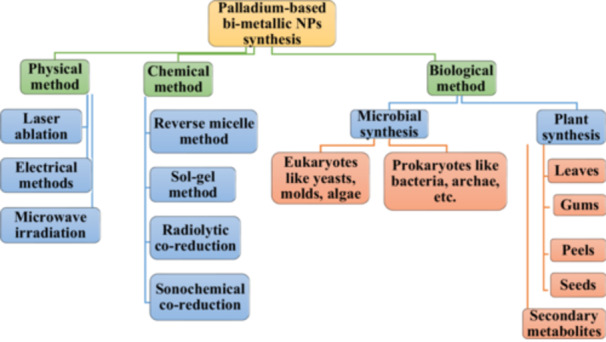
General schematic diagram of palladium‐based bimetallic nanoparticles synthesis types.

Biogenic NPs are produced by biological mechanisms and are more flexible for biomedical and other applications. NP synthesis is more time‐consuming, expensive, and needs hazardous chemical reagents than other physical or chemical processes, limiting its usefulness. The biological methods of NP synthesis are generally done in two ways: first, by using microbial flora, and second, from plant extracts. Biological approaches are also referred to as ‘green synthesis’ methods. Green synthesis produces NPs without the use of costly and toxic chemicals, and the end products are likewise more environmentally friendly. Biogenic NPs can be made more readily and cheaply, and the toxicity of the various metals or chemicals utilized in the synthesis can be avoided (Figure 5).

#### 5.3.1 Microbial synthesis using eukaryotes and prokaryotes

**Figure 6:**
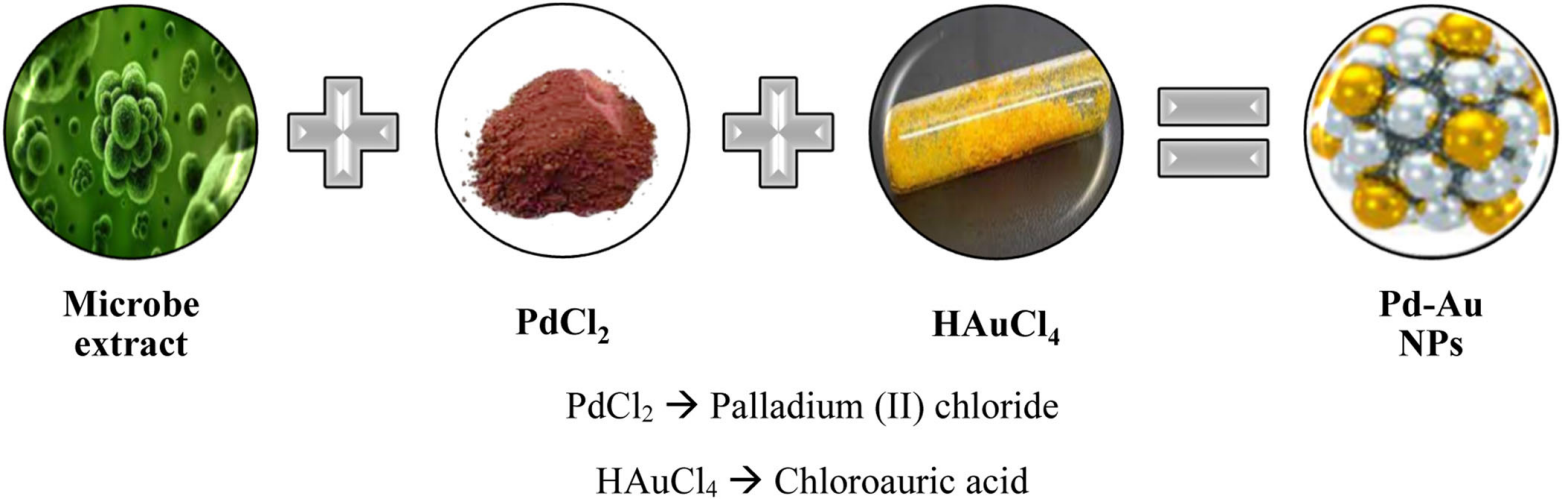
Graphical representation on synthesis of Pd‐Au nanoparticles using beneficial microbial isolates.

Bacteria, fungi and yeasts are the most commonly used microorganisms for NP generation, either intracellular or extracellular. In the presence of cellular enzymes, the intracellular mechanism involves the transport of ions into the cell of the microorganism to form NPs, whereas the extracellular mechanism involves the binding or adsorption of metal ions on the surface of the microbial cell, and reduction occurs in the presence of enzymes. The extracellular method is more acceptable, as the intracellular method requires downstream processing steps to recover the synthesized NPs. The recovery of NPs in the intracellular process comprises sonication to break down the cell wall as well as several centrifugation and washing stages to purify the NPs. Many scientists have been drawn to investigate the process involved in biogenic NPs due to the interaction between inorganic compounds and biological species. Some heavy metal NPs are rarely synthesized by microbes because heavy metals such as mercury (Hg^2+^), cadmium (Cd^2+^), cobalt (Co^2+^), copper (Cu^2+^), nickel (Ni^2+^), lead (Pb^2+^), and zinc (Zn^2+^) are harmful to microorganisms. Metal NPs can thus be generated by metallophilic microorganisms with the genetic and proteomic components required to respond to a hazardous environment. Metal‐resistant gene clusters exist in microbes and aid in cell detoxification via a variety of methods such as complexation, an efflux system, or reductive precipitation. Magnetic NPs are gaining popularity due to unique features such as superparamagnetism and high coercive force. They are much more adaptive in the field of biological separation and biomedicines due to their microconfiguration. Magnetite (Fe_3_O_4_) and maghemite (Fe_2_O_3_) are biocompatible magnetic NPs that are used in a variety of fields. Magnetotactic bacteria are responsible for their production. Magnetotactic bacteria use an internal mechanism to manufacture magnetic particles known as bacterial magnetic particles (BacMPs), which exist as aligned chains within the bacterium. They operate like compass needles, propelling the bacterium through the oxygen gradient in aquatic conditions. They scatter easily in aqueous solutions because they are surrounded by the bacterium's organic membranes, which are made up of phospholipids and proteins. BacMPs formation is a multistep procedure. In the first phase, the cytoplasmic membrane invaginates, forming a vesicle that acts as a precursor of the BacMPs membrane. All of the vesicles are linked together in a linear chain by cytoskeletal filaments. The second stage is BacMPs biomineralization via transmembrane ion transporters that accumulate ferrous ions (Fe^2+^) into the vesicles. Finally, closely bound BacMPs proteins stimulate magnetite crystal nucleation and govern their shape. Despite the fact that microorganisms are utilized for NP synthesis in a less expensive, effective and narrow‐sized dispersion, as well as in environmentally acceptable biocompatible goods, they have several limits. Culturing of microbes is time‐consuming. Controlling size distribution, form and crystallinity is difficult. The dispersibility of microorganism‐produced NPs is not uniform, resulting in polydispersibility. Production is moving at a slower pace due to the fact that difficulty in selecting the appropriate microorganism strains, difficulty in optimizing reaction parameters such as pH, temperature and incubation time, as well as metal ion concentration. Implementation of these biological techniques for large‐scale production and commercial applications is difficult. Omajali et al. (2019) [8] synthesized palladium/ruthenium (bio‐Pd‐Ru) core‐shell NPs from *Bacillus benzeovorans* and act as bio‐derived catalysts. The bio‐NPs were characterized using various electron microscopy techniques and high‐angle annular dark field (HAADF) analyses, which revealed two NP populations (1–2 nm and 5–8 nm), with core‐shell in the latter. The 0.231 nm Pd/Ru NP lattice fringes corresponded to the (110) plane of RuO_2_. While surface characterization with X‐ray photoelectron spectroscopy (XPS) revealed the presence of Pd(0), Pd(II), Ru(III) and Ru(VI), bulk material X‐ray absorption (XAS) analyses verified the Pd speciation (Pd(0) and Pd(II)—corresponding to PdO), and identified Ru as Ru(III) and Ru(IV). The absence of Ru‐Ru or Ru‐Pd peaks suggested that Ru existed solely in surface‐localized oxide forms (RuO_2_ and RuOH). Pd‐Ru alloying was not identified using X‐ray diffraction (XRD) patterns. The conversion of 5‐hydroxymethyl furfural (5‐HMF) to the fuel precursor 2,5‐dimethyl furan (2,5‐DMF) was investigated in preliminary catalytic investigations. Excellent loading (9.7 wt% Pd, 6 wt% Ru) and low loading (2.4 wt% Pd, 2 wt% Ru) bio‐derived catalysts displayed excellent conversion efficiency (95%) and selectivity of 63% (80% better than bio‐Ru NPs), respectively. These materials have a bright future as efficient low‐cost biofuel catalysts (Figure 6).

#### 5.3.2 Green synthesis using plant extracts: A new approach for the synthesis of BNPs

Phytonanotechnology opens up new possibilities in the field of NP synthesis because it is an eco‐friendly, simple, stable, fast and low‐cost process. Aside from these benefits, phytonanotechnology generates biocompatible NPs, the procedures are scalable, and, most significantly, no harmful chemicals are used as reducing agents. Generally, the universal solvent water serves as both a medium and a reducing agent. It is also known as a green synthesis approach because it may be done in a safer and simpler manner without the use of any chemicals. In green synthesis, the plant or parts of plants can be directly extracted in an aqueous medium. It can then act as a reducing, capping or stabilizing agent in the synthesis of metal NPs. Different functional groups such as phenolic or alcoholic groups, carboxylate groups, and so forth, are involved in the reduction, capping, synthesis and stabilization of NPs prepared by the green synthesis method. Green synthesis easily overcomes the constraints of traditional biological approaches, such as the use of microorganisms.

**Figure 7:**
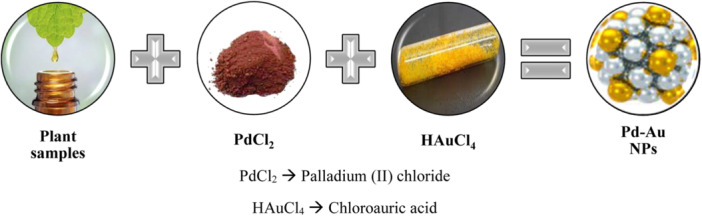
Graphical representation on synthesis of Pd‐Au nanoparticles using plant samples.

When compared to procedures aided by bacteria and fungus, plant‐mediated production of core‐shell NP is favourable. Plants are superior to microorganisms for NP synthesis due to their low handling costs, vast production volume, safety and so on. Various phytochemicals or secondary metabolites having functional groups such as COOH, C═O, NH_2_, SH and OH, such as polyphenolic compounds, flavonoids and terpenoids, give reduction and stabilizing activities to produced NPs. Various BNPs, such as Au‐Ag, Au‐Pd, Au‐Pt, Ag‐Cu and Fe‐Pd, have been synthesized utilizing plant biomass from *Aegle marmelos, Cacumen platycladi, Carica papaya, Opuntia ficus‐indica* and *Camellia sinensis*, among others. The various plant part extracts are employed here to synthesize metal NPs. Different mechanisms work for the production, reduction, capping and stability of metal NPs based on the chemical components and functional groups present in the plant part extract. The green synthesis of metal NPs is a bottom to a top method and different plant parts such as leaves, bark, stems, twigs, seedlings, fruits, pericarp, seeds, latex, gums, tissue cultures and secondary metabolites are utilized to prepare MNPs and BNPs in a safer, eco‐friendly, cost‐effective and easier way. It has been discovered that NPs created using the green approach are more stable than those prepared using other physical or chemical procedures (Figure 7).

(1) *Leaf extracts*
  Many publications have cited the production of Ag, Au and Pd NPs with various biological and medicinal functions. Carbohydrates, proteins, polyphenols and some vitamins act as reducing, capping and stabilizing agents in the creation of metal ion precursors, which are then converted into metal NPs. The morphology of the NPs, such as their size and shape, is determined by the concentration of metal salts and the length of time the extract is present in the reaction media. Scientists were inspired to create BNPs after successfully synthesizing MNPs. Mohan et al. (2021) [9] studied dual probes of Ag‐Pd bimetallic NPs facilely synthesized by green process using *Catharanthus* leaf extract on textile dye removal and free radical capability. Ag‐Pd BNPs were synthesized by greener way using *Catharanthus* leaf extract. The photocatalytic activity of the Ag/Pd BNPs was employed to degrade the safranin textile dye, and the free radical scavenging property was tested using the DPPH assay. Biogenic reduction was used to create Ag/Pd BNPs from silver nitrate and palladium chloride precursors, which were aided by CRL extract. The SEM/transmission electron microscopy (TEM) picture was used to assess the morphology and particle size of the produced Ag‐Pd BNPs. The particle size was determined to be between 15 nm and 30 nm. The photocatalytic activity of Ag/Pd BNPs on safranin O textile dye achieved 98% maximum dye degradation for 40 min, and the antioxidant activity of Ag/Pd BNPs was also tested using the DPPH assay. The free radical scavenging efficiency of extract and extract with Ag/Pd NPs was calculated and found to be 48.2% and 70.2%, respectively, indicating that the Ag/Pd NPs have 2.34 times the scavenging activity of *Catharanthus* leaf extract.
  Olajire et al. (2020) [10] performed green synthesis of bimetallic Pd_core_Au_shell_ NPs for enhanced solid‐phase photo degradation of low‐density polyethylene film using *Ananas comosus* leaf extract as a reducing agent. Using *A. comosus* leaf extract as a reducing agent, bimetallic core/shell Pd/Au (Pd_core_Au_shell_) NPs were produced from aqueous solutions of Pd (II) and Au (III) species. Fourier transform infrared spectroscopy (FTIR), ultraviolet visible absorption (UV‐Vis), high‐resolution TEM (HR‐TEM), energy dispersive X‐ray (EDX) and X‐ray diffraction (XRD) were used to characterize the as‐prepared NPs. An UV‐Visible spectroscopy revealed the bimetallic Pd_core_Au_shell_ structure, and the surface plasmon resonance (SPR) band occurred at roughly 517 nm, confirming the creation of an Au shell layer on the surface of pre‐formed Pd NPs. The bimetallic Pd_core_Au_shell_ NPs have particle sizes ranging from 2.06 nm to 28.59 nm, with an average particle size of (13.15 ± 6.22) nm, and they formed in face‐centred cubic (FCC) symmetry. The EDX analysis supports the bimetallism of Pd_core_Au_shell_ NPs, with each metal present in a 1:2 ratio. Using the solid‐phase breakdown of LDPE film, the photocatalytic potential of as‐synthesized Pd_core_Au_shell_ NPs was examined. After 24 h of solar light irradiation, the LDPE film with 1.0% Pd_core_Au_shell_ NPs deteriorated at a rate of 55.8 ± 5.9 compared to 8.6 ± 0.7 for pure LDPE. However, after 24 h in the dark, the degradation value of LDPE film with 1.0% Pd_core_Au_shell_ NPs was 1.90 ± 0.03. The NPs’ long‐term viability was demonstrated by their capacity to be reused in the photocatalytic degradation reaction for up to five consecutive cycles with no noticeable loss in catalytic efficiency. The NPs’ stability was demonstrated by SEM characterization before and after the degrading reaction. The current study for the polymer industry suggests developing eco‐friendly photodegradable plastic by including Pd‐Au NPs into the polymer matrix as a means of solving plastic pollution concerns.
  Minal et al. (2020) [11] performed laboratory analysis of Au‐Pd BNPs synthesized with *Citrus limon* leaf extract and its efficacy on mosquito larvae and nontarget organisms. The present investigation provides novel findings on the synthesis of Au and Pd (Au‐Pd) BNPs using an eco‐friendly and nontoxic aqueous leaf extract of the plant *Citrus limon*. The toxicity of BNPs on the larvae of disease vectors such as *Anopheles stephensi* and *Aedes aegypti* mosquitoes was investigated. The predation effectiveness test was performed on nontarget invertebrates such as natural predatory nymphs of dragonfly and damselfly. With the development of the SPR bands, the results of material characterization by UV‐Vis spectroscopy validated the synthesis of Au‐Pd BNPs. The presence of functional groups comprising large levels of nitro compounds and amines on the surface of BNPs is revealed by FTIR spectroscopy. The presence of spherical polydispersed Au‐Pd BNPs in the sample is revealed by TEM. The XRD pattern revealed the semi‐crystalline form of the sample, while DLS and ZP analyses revealed changes in the hydrodynamic size and surface potential during 0th, 24th, 48th and 72nd hour of synthesis. After 24th, 48th and 72nd hour of exposure, the Au‐Pd BNPs Bioassay provided effective lethal doses (LC_50_) against the I–IV instar larvae of *Anopheles stephensi* and *Aedes aegypti*. The larvicidal bioassay LC_50_ was performed to examine its influence on the predation efficiency of the selected nymphs, which exhibited increased predation from 40 h to 48 h of exposure when compared to the negative control. As a result, we conclude that the Au‐Pd BNPs bioassay demonstrates harmful mosquito larvicidal activity at the specified dose while having no deadly effect on the predatory nontarget aquatic invertebrate insect predation efficiency.
  Amalia et al. (2020) [12] performed green synthesis of Au‐Pd core‐shell NPs using orange peel extract through two‐step reduction method and its formaldehyde colorimetric sensing performance. The production of Au NPs was indicated by a significant visible absorbance at 534 nm at an optimum water/OPE ratio of 16.67, while the formation of Au‐Pd NPs was indicated by the removal of the peak at 534 nm. IR spectroscopy revealed that the −OH groups of OPE play the most important role in NP production, while TEM images revealed a core‐shell structure of the Au‐Pd NPs with an average core diameter of 40 nm and an average shell thickness of 7 nm. A two‐step simple and environmentally friendly method for creating Au‐Pd core‐shell nanoparticles (Au‐Pd NPs) has been devised. HAuCl_4_ and H_2_PdCl_4_ were used as precursors, with orange peel extract (OPE) acting as a reducing and stabilizing agent. The Au core NPs (Au NPs) were initially produced using OPE, and then the Pd shell was formed by reducing PdCl_4_^2−^ solution. The core‐shell NPs changed colour from light to dark brown in the presence of varied formaldehyde concentrations. The linearity of the absorbance responses from the formaldehyde concentration range of 36.3 mM to 3.63 M (*R* ^2^ = 0.991) with an estimated limit of detection of 304.9 mM indicated that the produced NPs are promising for formaldehyde sensing.
(2) *Peel extracts and gum extracts*
  Many citrus fruits’ aqueous extracts are employed in the creation of metal NPs. Ascorbic acid and essential oils (EOs) found in fruit peel aid in the reduction and stability of certain metal precursors. Gum extracts from various plants are also suitable candidates for green production of Ag, Au and Pt NPs and exhibit antibacterial action. Velpula et al. (2021) [13] synthesized bimetallic nanocomposites (Ag‐Au, Ag‐Pd and Au‐Pd) synthesis using gum kondagogu, a natural biopolymer and their catalytic potentials in the degradation of 4‐nitrophenol. BNPs are composed of two distinct metal elements and, as a result of their synergistic activities, have better mechanical and catalytic efficacies than MNPs. In this study, various bimetallic Ag‐Au, Ag‐Pd and Au‐Pd NPs were produced in a straightforward and cost‐effective manner using a natural biopolymer gum kondagogu (GK) as a reducing and capping agent. The UV‐Vis spectroscopy of the generated BNPs indicated that each nanocomposite has a different surface plasmon resonance band (SPR). The average particle size of Ag‐Au, Ag‐Pd and Au‐Pd BNPs was 23.10, 21.76 and 23.94 nm, according to transmission electron microscopy studies. The surface morphology and functional groups on the gum matrix of GK‐BNPs were investigated using XRD and FTIR. The capacity of bimetallic nanocomposites to catalyse the reduction of 4‐nitrophenol (4‐NP) to 4‐aminophenol was investigated in the presence of NaBH_4_. The rate constants for Ag‐Au, Ag‐Pd and Au‐Pd NPs were calculated using kinetic studies and were 0.31, 0.39 and 0.28 min correspondingly. The catalytic efficiencies of three bimetallic nanocomposites were Ag‐Pd > Ag‐Au > Au‐Pd in that order. This study examines the catalytic potentials of three distinct bimetallic nanocomposites in the reduction of 4‐NP, a pollutant, as well as the impact of their synergistic property.
(3) *Seed extracts*
  Numerous investigations have been published on the production of Ag and Au NPs and nanocomposites from aqueous extracts of the seed powders of *Elaeocarpus ganitrus* Roxb. (Rudraksha), *Jatropha curcas, Macrotyloma uniflorum, Trigonella foenum graecum* (fenugreek), *Artocarpus heterophyllus* and other plants. Ag and Au NPs with various morphologies exhibit antimicrobial and antibacterial activity. Aygun et al. (2023) [14] investigated the highly active Pd‐Pt BNPs synthesized by one‐step bioreduction method: Characterizations, anticancer, antibacterial activities and evaluation of their catalytic effect for hydrogen generation. Metallic nanoparticles (MNPs) have numerous medical and technological applications. Because of their qualities, bimetallic NPs, which are metallic nanoparticles, are of tremendous interest. To obtain bimetallic NPs, a new green synthesis approach was developed. This method of synthesis employs aromatic herbs. *Nigella sativa* is one of the plants used for green synthesis, and it has a special niche among plants for use as medicine. The synthesis of Pd‐Pt bimetallic nanoparticles (Pd‐Pt NPs) and the catalytic, antibacterial and anticancer properties of Pd‐Pt NPs generated via green synthesis utilizing *Nigella sativa* seed extract are reported in this paper. FTIR, TEM, X‐ray diffraction (XRD) and UV‐Vis spectrometry techniques were used to analyse the produced Pd‐Pt NPs. Sodium borohydride (NaBH_4_) hydrolysis studies were used to investigate the catalytic activity of Pd‐Pt NPs. Turnover Frequency (TOF), activation energy, entropy and enthalpy values were determined to be 1664.76 h^−1^, 13.93 kJ/mol, 119.02 kJ/mol and 11.43 kJ/mol, respectively. Pd‐Pt NPs were discovered to be highly effective hydrogen generation catalysts. Antibacterial activity of Pd‐Pt NPs (200 g/mL) against *Escherichia coli, Staphylococcus aureus, Methicillin‐resistant Staphylococcus aureus* and *Bacillus subtilis* bacteria was determined to be 57.58%, 64.42%, 48.68% and 58.77%, respectively. Furthermore, the cytotoxic effects of Pd‐Pt NPs and MTT against human breast cancer cell line (MDA‐MB‐231), human endometrial carcinoma cell line (Ishikawa, ISH), human cervical cancer cell line (HeLa), L929‐murine fibroblast cell line test and IC_50_ values were determined. Pd‐Pt NPs had IC_50_ values of (9.1744 ± 1.566) µg/mL, (12.2431 ± 1.132) µg/mL and (18.1963 ± 1.730) µg/mL against MDA‐MB‐231, ISH and HeLa cancer cell lines, respectively. In healthy L929‐murine fibroblasts, no cytotoxic impact was found. The green production of Pd‐Pt NPs was shown to have substantial advantages over chemical methods. The biogenic Pd‐Pt NPs generated in this study point to the development of bio‐based bimetallic catalysts with strong catalytic performance to reduce pollution. Fahmy et al. (2021) [15] performed green synthesis of Pt and Pd NPs using *Peganum harmala* L. seed alkaloids: biological and computational studies. The work describes a simple and environmentally friendly approach for producing Pt and Pd NPs (Pt NPs and Pd NPs) from the seed alkaloid fraction of *Peganum harmala*. The potentials of the synthesized Pt NPs, Pd NPs and Pt‐Pd NPs were respectively (11.2 ± 0.5), (9.7 ± 1.2) and (12.7 ± 2.1) mV. The production of spherical‐shaped Nps with smooth edges was detected by TEM. TEM investigation revealed that the mean diameters of the generated Pt NPs, Pd NPs and Pt‐Pd NPs were (20.3 ± 1.9), (22.5 ± 5.7) and (33.5 ± 5.4) nm, respectively. UV‐Vis spectroscopy, X‐ray diffraction (XRD) and FTIR were used to confirm the bio reduction of the nanoparticles, and thermal gravimetric analysis (TGA) was used to evaluate their organic content. The antioxidant activity of the Pt‐Pd NPs mixture was (843.0 ± 60) M Trolox equivalent (TE)/mg NPs, which was higher than that of the individual Pt NPs ((277.3 ± 13.5) M TE/mg NPs) and Pd NPs ((167.6 ± 4.8) M TE/mg NPs). In addition, the Pt‐Pd NPs exhibited significant cytotoxic activities against lung cancer (A549) and breast adenocarcinoma (MCF‐7) cells, IC_50_ of 8.8 and 3.6 µg/mL, respectively, as compared to Pt NPs (IC_50_ of 10.9 and 6.7 µg/mL, respectively) and Pd NPs (IC_50_ of 31 and 10.8 µg/mL, respectively), and compared to carboplatin (IC_50_ of 23 and 9.5 µg/mL, respectively). Additionally, molecular docking investigations were carried out to investigate the biogenic NPs’ potential anticancer and antioxidant actions. Pt NPs, Pd NPs and their mixture inhibited cysteine proteinase, indicating that they have excellent anticancer efficacy but only moderate antioxidant activity. Finally, Pd‐Pt NPs synthesized from harmala seed alkaloid fraction demonstrated potential as effective antineoplastic agents.
(4) *Secondary metabolite extract*
  Secondary metabolites are also a useful starting point for the green synthesis of Ag and Au NPs with high anti‐cytotoxic action. Aside from the examples given above, additional portions of the plants are used for aqueous extraction and then subjected to green synthesis of various metal NPs. BNPs are often synthesized through a stepwise reduction or co‐reduction of metal ions, which results in alloy, mixed or core‐shell BNPs. The metal salts are applied one by one in the first technique to extract so that the metal precursor forms nuclei on which the second metal precursor produces a core‐shell BNP. When metal precursors are added to the extract at the same time, the reduction of the metal ion that occurs earlier and that occurs later is determined by its reduction potential. This method yields either an alloy or a mixed form of BNP. Rashidi et al. (2019) [16] performed green synthesis of Pd NPs supported on modified nonpareil almond shell (NAS) using almond hull extract: a beneficial nanocatalyst for convenient reduction of organic dyes. Pd NPs were created utilizing Nonpareil almond husk as a reducing and stabilizing agent in this study, without the need of any harmful solvents or capping agents. Two distinct approaches were used in this protocol to immobilize Pd NPs on the surface of green‐waste NAS, and modified NAS was obtained as an environmentally friendly support. UV‐Vis spectroscopy, FTIR, X‐ray diffraction (XRD), field emission scanning electron microscopy (FE‐SEM), energy‐dispersive X‐ray spectroscopy (EDS), Brunauer–Emmett–Teller (BET) and TEM were used to characterize the green nanocatalyst. The Pd NPs immobilized on modified NAS are spherical particles with an average size of less than 20 nm and no aggregation, according to TEM images. To study the catalytic activity of green produced nanocatalyst, it was employed in the reduction of methylene blue (MB), rhodamine 6G (R6G) and methyl orange (MO) at room temperature. According to the results, the modified nanocatalyst displayed a high catalytic activity in the reduction of various organic dyes. Furthermore, the nanocatalyst may be simply recycled and reused multiple times without losing activity.

## 6 CHARACTERIZATION OF PD‐BASED BIMETALLIC NPS

**Figure 8:**
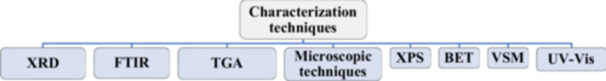
Different characterization techniques used for palladium‐based bimetallic nanoparticles.

Identifying and studying the physicochemical properties, structure details, purities and dopants of biosynthesised NPs is very important as the structure, size and shape of NPs can significantly affect their performance and properties. In fact, determining the physicochemical characteristics of NPs helps to effectively understand the relationship between these characteristics and their performance. From previous studies, the antimicrobial efficiency of Ag NPs with smaller sizes seems to be more than that of the larger sizes. Describing the behaviour and structure of green synthesized NPs is quite difficult due to the existence of various macromolecules in their extract which participate in their own structure. In the following sections, common physicochemical characterization techniques of NPs are described briefly [17] (Figure 8).

### 6.1 Spectroscopic analysis

UV‐Vis spectroscopy analysis is widely used for the primary detection of various types of NPs that can absorb electromagnetic radiation in the UV‐Vis spectral region. For example, the UV‐Vis range of Pd‐based BNPs is around 250–750 nm range.

### 6.2 Microscopic analysis

The size, morphology and distribution of nanomaterials have been investigated using microscopic techniques such as analysis, scanning electron microscopy (SEM), field emission SEM (FESEM), atomic‐force microscopy (AFM), TEM and HR‐TEM, and AFM. AFM and SEM images are ineffective for studying the structure of core‐shell NPs because they characterize the surfaces. Knowing about the core in SEM images is difficult, whereas TEM images are very useful for studying the structure of core‐shell NPs because they can measure the thickness and spacing between the core and the shell/shells. Energy dispersive spectrometry (EDS) is an add‐on for electron microscopy instruments (TEM and SEM). EDS is a powerful method for determining the chemical nature of the core and shell, and it has revealed the distribution of elements in the studied samples. X‐ray diffraction (XRD) scattering and XRD analysis were used to examine the crystal structure and phase purity of the synthesized NPs (crystal structure refers to the ordered arrangement of atoms).

### 6.3 FTIR

The surface modification of NPs, the load‐drug and overlay functionalities, the types of functional groups and biomolecules responsible for capping and effectively stabilizing NPs, the presence of shells in core‐shell NPs, the verification of the band between the two layers of the shell in core double shell NPs, and the qualitative and quantitative identification of the molecular structure of organic compounds in the NPs structure—particularly in the structure of hollow‐core/porous‐shell materials or core‐shell NPs—can all be identified using FTIR. FTIR analysis is done in the range of 500–4500 cm^−1^ for Pd‐based BNPs.

### 6.4 TGA

Through the use of thermal analysis, or TGA, the mass of NPs is tracked over time as temperature changes, typically between 25°C and 800°C. The TGA can also test the properties of the core‐shell NPs’ oxidation resistance. Drug structure, hydration, drug absorption efficiency in mesoporous NPs and hybrid NPs’ magnetic performance can all be ascertained with the help of this analysis. It is possible to conduct TGA measurements in a variety of atmospheres, including argon, hydrogen, air and ozone. With the TGA analysis in ozone, we can determine how many organic molecule residues are present in the structure of NPs. The breakdown of biomolecules in the NPs’ structure, which is significant when you consider that the biomolecules in the extract serve as both capping and reducing agents, is the cause of the mass loss in green synthesized NPs at high temperatures. Catalytically active, Pd‐based NPs have been synthesized with average diameters ranging from 1.9 nm to 7.4 nm.

### 6.5 Vibrating sample magnetometer (VSM)

The magnetic characteristics of NPs can be investigated using VSM. It is now significant and feasible in biology, medicine, and industry applications due to the preservation of magnetic properties, attainment of higher magnetic properties, and assessment of the magnetic performance concerning the core‐shell NPs in comparison to the single structured NPs.

### 6.6 XPS

To understand the chemical and physical behaviours of core‐shell NPs, it is imperative to determine their oxidation status in catalytic systems and gas detection sensors. Through the use of XPS analysis, a method for analysing surface elements with a nanometre sampling depth, the surface oxidation of these NPs has been examined. It can additionally determine the atomic ratio in NPs with heterogeneous structures and provide chemical information for specific elements, such as distinguishing between the sulphate and sulphide forms of sulphur, which is required to understand the morphology of NPs core‐shell.

### 6.7 Brunauer‐Emmett‐Teller

Accurate assessment of surface area, volume and pore distribution is crucial for describing pharmaceutical materials made of polymers and NP coatings. The form and location of the cavities in relation to one another within the NPs texture, as well as the structure of the porous, were all determined using BET analysis. The amount of drug loaded in NP‐based targeted drug delivery systems, as well as controlling the release of the loaded drug, absorption, storage, catalysis and other activities, are all significantly influenced by the hybrid NPs’ surface specific area, surface characteristics and pore volume.

Other characterization techniques such as XRD analysis, zeta‐potential, SEM and TEM microscopy, NMR‐ESR resonance and so forth can be used to view the size, range, distribution and structure of Pd‐based BNPs.

## 7 BIOMEDICAL APPLICATIONS OF PD‐BASED BNPS

BNPs have several applications in medicine, including diagnostic (bio‐imaging), treatment (cancer therapy), and preventative (antimicrobial, antioxidant, and antidiabetic medication delivery). The extremely magnetic features of Au‐Fe and Ni‐Co BNPs make them excellent for use as a contrast agent for computed tomography (CT) and magnetic resonance imaging (MRI) diagnosis/prognosis imaging, as well as a theranostic agent for tumours. Similarly, Cu‐Fe has been employed for increased chemo dynamic therapy, Pd‐Pt, Au‐Co, Au‐Pd, Ag‐Cu and Au‐Pt for cancer therapies and anticancer activities, and Au‐Bi for tumour cell suppression. BNPs are widely employed in preventative medicine, with many of them serving as antibacterial agents, antioxidants, antidiabetic, anti‐Alzheimer, anti‐inflammatory and drug delivery agents. These are often utilized in controlled medication delivery systems that aid in the therapy of a sick condition in the biological environment. The release rates of the entrapped pharmaceuticals are regulated in the nanosystems to meet the intended therapeutic parameters, resulting in drug release at a predefined rate over time. This leads in the preservation of a therapeutic window for a set amount of time. They are suitable carriers in targeted drug delivery systems for delivering entrapped therapies at the illness site with decreased off‐target side effects, enhancing cellular absorption of poorly water‐soluble medicines, resulting in enhanced bioavailability (Table 3).

**Table 3:** Past works of palladium‐based bimetallic nanoparticles

NPs	Plant source	Microbial source	Characterization	Pathogen studied	Activity studied	Reference
Pd/Ru bimetallic nanoparticles	–	*Bacillus benzeovorans*	HAADF, XPS, XAS, XRD	–	Biofuel catalysts	Omajali et al. [8]
Pd_core_Au_shell_ nanoparticles	*Ananas comosus*	–	FTIR, UV‐Vis, HR‐TEM, EDX, XRD, SPR and SEM	–	Photodegradation of LDPE	Olajire et al. [10]
Au‐Pd bimetallic nanoparticles	*Citrus limon* leaf	–	UV VIS, FTIR, TEM, XRD, DLS and ZP	–	Larvicidal activity of *Anopheles stephensi* and *Aedes aegypti* mosquitoes	Minal et al. [11]
Gold‐palladium core‐shell nanoparticles	Orange peel	–	IR spectroscopy, UV‐Visible, XRD, TEM, EDS and FTIR	–	Formaldehyde colorimetric sensing performance	Amalia et al. [12]
Pd‐Pt	*Nigella sativa* seed	–	FTIR, TEM, XRD, UV‐Vis	*E. coli*, *S. aureus*, MRSA, and *B. subtilis*	Anticancer activity against breast cancer cell line, endometrial carcinoma cell line, cervical cancer cell line and catalytic effect for hydrogen generation	Aygun et al. [14]
Pt‐Pd NPs	*Peganum harmala* L.	–	UV‐Vis, XRD, FTIR, TGA, TEM	–	Cytotoxic activities against lung cancer (A549) and breast adenocarcinoma (MCF‐7) cells	Fahmy et al. [15]
Silver chloride‐anchored palladium/gold bimetallic alloy nanoparticles	–	*Bos taurus indicus* urine	XRD, FTIR, FESEM, EDS, XPS and particle size analysis and Zeta (ξ) potential	*S. aureus* and *E. coli*	FRAP Assay, catalytic nitroarenes reduction assay, dye degradation assay	Sarvalkar et al. [18]
Silver‐based palladium nanoparticles	*Hibiscus sabdariffa*	–	FTIR, UV‐Vis, XRD	*B. cereus, E. faecalis, E. coli, P. aeruginosa, B. subtilis* and *C. albicans*	–	Karimi et al. [19]
Pt‐Pd NPs	*Hibiscus sabdariffa*	–	UV‐Vis, TEM, AFM, XRD	–	Antioxidant activity, antibacterial activity, and lipid peroxidation inhibition activity	Seckin et al. [20]
Core‐shell Cu/Pd nanoparticles	–	Natural chitosan and applied to glucose detection	TEM, XPS, XRD, HAADF‐STEM, SEM	–	Glucose detection	Wang et al. [21]
Au‐Pd bimetallic nanoparticles	Fenugreek seed polysaccharide	–	SEM, TEM, UV‐Vis, XRD, FTIR and XPS	–	Catalytic application	Mallikarjuna et al. [22]
Pd/Fe_3_O4 nanocomposite	*Hibiscus tiliaceus* L.	–	FTIR, UV‐Vis spectroscopy, double‐beam spectroscopy, XRD, FESEM and EDS	–	Reductive catalysis of Cr(VI) and nitro compounds	Nasrollahzadeh et al. [23]

Abbreviations: EDS, energy‐dispersive X‐ray spectroscopy; EDX, energy dispersive X‐ray; FTIR, Fourier transform infrared spectroscopy; HAADF, high‐angle annular dark field; HR‐TEM, high‐resolution transmission electron microscopy; SPR, surface plasmon resonance; TEM, transmission electron microscopy; UV‐Vis, ultraviolet visible; XAS, X‐ray absorption; XPS, X‐ray photoelectron spectroscopy; XRD, X‐ray diffraction.

### 7.1 Antioxidant activity

Plant‐based phytochemicals, particularly polyphenols (such as flavonoids and phenolic acids), have been extensively researched. Free radical defence, immune system bolstering and inflammation reduction are all facilitated by flavonoids. They are in charge of the antioxidant properties of plants. Numerous hydroxyl groups connected to the aromatic ring's carbon atoms are present in these naturally occurring compounds. Many phytochemicals are found directly under the bark or in the outer leaves. They are unique to each and every plant, including individual cells. On top of that, some authors think that the combination of phytochemicals and the NP of noble metals lowers the risk of colon, breast or lung cancer. This is due to antioxidants, which fight the destructive effects of free radicals. There are two categories of antioxidant activity mechanisms:

i. HAT is a method of donating a hydrogen ion from a stable molecule, allowing the antioxidant to eliminate reactive oxygen species (ROS).
ii. The ability of antioxidants to transfer an electron and reduce specific molecules and compounds is known as a single electron transfer (SET). As a result, it is concluded that antioxidants found in plants are responsible for the green synthesis of metal NPs or metal oxides due to their ability to reduce or chelate metal ions while also acting as NP stabilizers.

Specific compounds’ antioxidant activity can be demonstrated in a variety of ways. One widely used method is the discoloration of the stable radical DPPH (2,2‐diphenyl‐1‐picrylhydrazyl). According to the latest research, BNPs have twice the antioxidant activity of MNPs. This feature, which is caused by H^+^, has the ability to break free radical chains. One example involved the synthesis of Pt‐Pd BNPs from *Peganum harmala* by Fahmy et al. [15]. The antioxidant activity of Pt‐Pd BNPs was (843.0 ± 60) µM TE/mg NPs, while Pt NPs were (277.3 ± 13.5) µM TE/mg NPs and Pd NPs were (167.6 ± 4.8) µM TE/mg NPs. It has anticancer properties against lung cancer (A549) and breast cancer (MCF‐5), in addition to its antioxidant activity.

### 7.2 Antimicrobial activity

**Figure 9:**
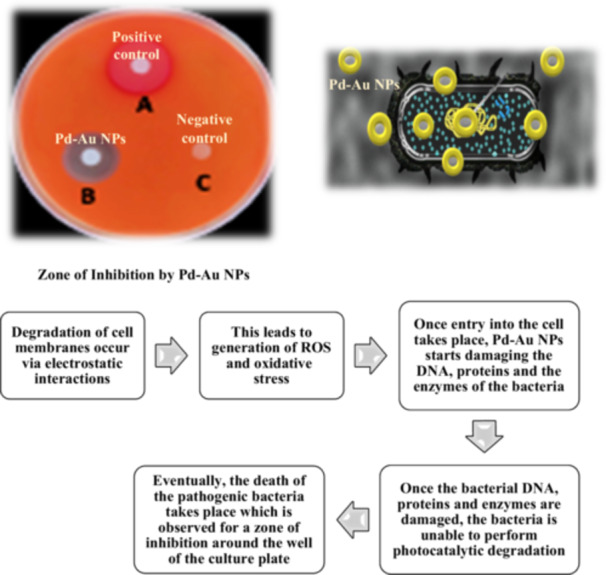
Represents the antimicrobial activity of Pd‐Au NPs nanoparticles and its mechanism.

The rapid development of drug‐resistant infectious illnesses is a global concern to human health today, causing morbidity and mortality. If this trend continues at its current rate, more people will die by 2050 because drug‐resistant infectious diseases are more fatal than cancer. Bimetallic NPs inhibit pathogenic bacterial development by disturbing bacterial membranes, producing ROS, damaging DNA and regulating protein function. The synergistic antimicrobial efficiency of BMNPs advances nano medical solutions toward a fundamental understanding of existing and emerging diseases, diagnostics and therapy. BMNPs outperformed traditional antibiotic and antimicrobial treatments against numerous Gram positive and Gram negative bacteria and viruses due to their synergistic antimicrobial efficacy (Figure 9). Controlling bacterial biofilm formation of human pathogenic microorganisms that cause severe chronic lung, urinary tract and other nosocomial infections is critical. The antibacterial activity of PD‐based NPs (Pd‐based NPs) was recently identified, and the published data indicated that Pd‐based NPs have a significant potential for antimicrobial applications. These combinations of Pd‐based NPs can generate ROS, which induce cell membrane damage, DNA damage, protein denaturation, and electron transport disruption, ultimately leading to bacterial mortality. The antimicrobial activity of Pd‐based NPs is highly reliant on their size and structure. Pd nanoparticles (Pd NPs) combined with another metallic NPs (Ag, Au, Pt, etc.) produced using biogenic techniques also demonstrated antibacterial activity. Pd NPs and Ag NPs, for example, greatly improve the bactericidal acidity of the produced composites (hydroxyapatite/chitosan/Ag‐Pd), and these scaffolds can be employed in dental procedures. The biosynthesized Pd‐Ag‐decorated rGO nanostructures by Mallikarjuna et al. (2021) [24] exhibited remarkable antimicrobial activity, effectively rendering 96% of *Escherichia coli* cells inactive over a 150‐min exposure to visible light. For the purpose of developing alternative nanomaterial‐based drugs in the future, these biosynthesized Pd‐Ag‐decorated rGO nanostructures can be employed. For example, Pd‐based NPs produced using biomass waste from *Moringa oleifera* petals by Anand et al. (2016) [25] as a natural reducing and capping agent shown remarkable antibacterial action against *Enterococcus faecalis*. Combining Pd NPs with other metals could result in a novel composite with antibacterial capabilities.

### 7.3 Anticancer activity

Cancers result from the death of healthy cells combined with unchecked cell division and abnormal tissue growth. The number of cancer cases and deaths worldwide increased to 18.1 million in 2018. About 1,735,350 cancer cases were diagnosed and 609,640 people died from cancer in the United States alone in 2018. Humans began using surgery as a cancer treatment. Nowadays, we live in the age of chemotherapy and radiotherapy. First, chemical drugs are administered intravenously or orally, and once in the bloodstream, they target cancer cells and cause cell‐to‐cell death. Radiotherapy, on the other hand, uses an ionic radiation dose in the tumour location to cause DNA damage and cell death. Both techniques are frequently used together to improve treatment efficacy. Nonetheless, they both have important limitations, such as a unique effect on each patient and potentially harmful side effects such as anaemia, organ damage, hair loss, and vomiting, among others. Due to the limitations mentioned above in chemotherapy and radiation therapy, several new treatments have emerged in recent decades, such as immune therapy, hyperthermia, and gene therapy.

The application of nanotechnology in cancer treatment has grown in popularity because it allows us to overcome some of the current limitations of imaging techniques and drug delivery methods. In pharmacological administration and cancer imaging, BMNPs are used in conjunction with specific techniques. For example, Hariharan et al. (2019) [26] performed green approach synthesis of Pd‐TiO_2_ NPs: characterization, visible light active picric acid degradation and anticancer activity. *Aloe vera* gel is taken out of the *Aloe vera* plant's leaves in this, as was previously reported. In addition to the 10 millilitres of collected *Aloe vera* gel, 120 millilitres of distilled water were used. The above‐mentioned green solution was heated to 100°C for 30 min to produce a uniform *Aloe vera* aqueous solution. About 0.1 M titanium (IV) isopropoxide and 0.003 M palladium acetate were added drop wise to the aforementioned solution and vigorously stirred for an hour at 60°C to achieve a homogeneous mixture. The above homogeneous solution was heated in a hotplate at 80°C for an entire night to eliminate some undesired impurities from the sample. The resulting white powder was then annealed in a muffle furnace at 500°C for 5 h to produce Pd‐TiO_2_ (anatase) NPs, and the NPs were characterized using UV‐vis spectroscopy, HR‐TEM at 200 kV, XRD analysis, TEM imaging and Raman spectroscopy, as well as visible light active picric acid degradation and antioxidant activity. Using XRD analysis, which shows the XRD spectra of both the Pd‐TiO_2_ and pure TiO_2_ NPs, the produced pure and Pd‐TiO_2_ NPs were phase identified. Regarding Pd‐TiO_2_ NPs and pure TiO_2_, the observed peaks corresponded to JCPDS card number 21‐1272. The peaks were measured at 25.4°, 37.7°, 48.2°, 53.9°, 55.1°, 62.5° and 70.4°, respectively, and were located in the planes (101), (004), (200), (105), (211), (204) and (220).

Pd concentration was so low (0.003 M) that no diffraction peaks were visible. In addition, the intensity of Pd‐TiO_2_ was significantly lower than that of pure TiO_2_ because of the *Aloe vera* gel molecules present. Utilizing XRD spectra, the lattice parameters (*a* = 3.7842, *c* = 9.5141 [Å]) were established, and they demonstrated a reasonably good agreement with the anatase phase documented in the previous study. An energy mode analysis of green synthesized TiO_2_ NPs was performed using the Raman spectrum. According to the Raman study, factor group analysis was used to identify the six Raman active modes (A1g^+2^B1g^+3^Eg) of an anatase TiO_2_. The study found four Raman active modes at 146, 399, 514 and 636, which corresponded to (Eg), (B1g), (B1g) and (Eg), respectively. The peak broadening and shifts of Raman bands were attributed to particle size reduction. Furthermore, Pd‐TiO_2_ perfect energy modes with minuscule Pd dopant concentrations have been attained. The Pd NPs were easily visible as tiny dots covering the entire surface, in contrast to the observed TiO_2_ NPs, which lacked shape in the HR‐TEM image of the Pd‐TiO_2_ NPs. Additionally, the estimated size of the Pd NPs was found to be 11 nm. *Aloe vera* gel powder that has been calcined at 500°C has strong IR bands at 3411.2, 1615.8, 1423.6, 1059.7 and 660.5 cm^−1^, according to the FTIR spectrum. Pd‐TiO_2_ exhibits photocatalytic activity in the degradation of picric acid. Pd‐TiO_2_ catalyst (20 mg) was added to a 500 mg/L solution of picric acid. To attain absorption‐desorption equilibrium, the solution was constantly shaken and exposed to a mercury lamp (*λ* = 543 nm) in the dark. Every 5 min, 5 mL of the solution were removed and examined using UV‐Vis spectroscopy. Pd‐doped TiO_2_ outperformed pure TiO_2_ in terms of photocatalytic activity. Cell viability assays were used to evaluate the anticancer potential of Pd‐TiO_2_ NPs against A549 cell lines. Pd‐TiO_2_ and pure TiO_2_ were found to have IC_50_ values of 165 and 210 μg/mL, respectively, against A549 cells. When contrasted with pure TiO_2_, green‐synthesized Pd‐TiO_2_ showed stronger anticancer benefits. A low band gap value was the outcome of red shift absorption in the visible region in Pd‐TiO_2_. In conclusion, a green method was used to synthesize both pure and Pd‐TiO_2_ NPs. Observing the interaction between Pd metal dopants and pure TiO_2_ NPs is one approach to investigate the photocatalytic degradation of picric acid in water. As a capping material, naturally occurring *Aloe vera* gel has been used to reduce the toxicity of the picric acid pollutant. It was discovered that the picric acid degraded in roughly 70 min when exposed to visible light. It was also discovered that Pd‐TiO_2_ NPs exhibited increased anticancer activity by absorbing visible light, which in turn caused a greater quantity of electron transfers to cell lines and consequent cell death.

### 7.4 Bioimaging applications

BMNPs generated using chemical reduction processes have crucial biomedical implications in illness detection employing bio‐imaging (CT, MRI, PET). PTT is gaining popularity due to its promise in the treatment of cancer and infection. The optimum photothermal agent should have the following properties: water dispersion, compact size, uniform shape, high absorption in the near‐infrared (NIR) region, high photostability, high biocompatibility, targeting to cancer cells/bacteria and ease of cleaning from kidneys. The photo‐absorbers had to have substantial absorption in the NIR region (700–2500 nm), which is an ‘optical window’ with the least light scattering and absorption of tissue, so that the absorbed light could penetrate deeply into the tissue.

Due to their high photothermal conversion efficiency, photostability, diversity in shape and size, and significant absorptions in the NIR range, Pd NPs are currently developing as an efficient photothermal agent for PTT. Multiantibiotic resistance has become a major issue in modern medicine. Thus, the development of innovative therapies for the treatment of infection is critical. Pd‐based NPs (Pd‐based NPs) were chosen as a photothermal agent to embed into the photothermally responsive chitosan/polyvinyl alcohol membrane due to their good photothermal behaviour. The created membrane demonstrated great biocompatibility, high porosity, a high degree of swelling, high moisture retention and strong photothermal performance. Pd NPs are interesting candidates for cancer/infection photothermal therapy because of their biocompatibility, photostability, strong absorption in the NIR region, high photothermal conversion efficiency, size and shape diversification and cost‐effectiveness.

There are other studies that involved in the usage of Pd‐based BNPs such as antidiabetic activity, antibiofilm activity, in‐vivo studies, biosensing applications, larvicidal activity and so forth.

## 8 ADVANTAGES AND DISADVANTAGES OF PD‐BASED BNPS

BNPs, especially the Pd‐based BNPs, have various advantages over their monometallic counterparts, but however the powerful and potent their advantages are, they do come with several disadvantages that we need to look at them before considering [27] (Table 4).

**Table 4:** Advantages and disadvantages of palladium‐based bimetallic nanoparticles.

Advantages	Disadvantages
Bimetallic nanoparticles, especially the Pd‐based, have improved catalytic properties and can maintain their catalytic activity for up to six catalytic cycles.	Environmentally unfriendly due to usage of unfriendly chemicals and solvents.
Pd‐based BNPs have been used as antimicrobial agents and have been found to be more effective than single metallic nanoparticles due to their combination of the properties of two different metals.	In some methods, limited control over the morphology of the Pd‐based BNPs.
Pd‐based bimetallic nanoparticles are highly biocompatible and stable, and have relatively low toxicity.	Some drawbacks of Pd‐based BNPs include the movement of material from the deposited substrates for further evaluation.
Palladium bimetallic nanoparticles can be used for drug delivery, especially for treating cancer.	Instability of the Pd‐based bimetallic NPs can sometimes occur.
Palladium nanoparticles that are combined either with gold and silver can be used for in vitro and in vivo photothermal cancer therapies.	The metal used as the core in the metal‐based NPs can cause toxicity in the human body.
They have high catalytic efficiencies when combined with metals such as gold, platinum, iron, and so forth.	There are chances of contamination during synthesis.
Provision of more efficient, cost‐friendly and better performance than its mono‐metallic counterparts.	Challenges in their synthesis can also happen.
Potency to reduce the side effects of conventional drugs.	These nanoparticles can have an adverse effect on cell viability, cell proliferation, and other biological mechanisms.
Can be used for the production of biomasks, sanitizers, biosensors, and so forth.	The metal precursor for the synthesis can also be unavailable and the cost of a palladium precursor is expensive when compared to other metal precursors.

## 9 CONCLUSION

The state‐of‐the‐art presents the potential of BNPs in several domains of biomedicine. BNPs perform admirably in antibacterial, anticancer and drug development research, and can be investigated in vitro or in vivo, just like their mono‐metallic counterparts. They are also researched in plants for heavy metal stress, plant growth development and other purposes. These NPs can be produced using any means, including chemical, hydrothermal and even biogenic methods. Plants and bacteria have the potential to synthesize BNPs without harming the environment, resulting in new products that can be studied or employed for the production of bio‐masks, effective hand sanitizers, medications and even vaccine development. The scope of these nanomaterials in medical applications is vast, and the future of nanotechnology is bright with the growth of technology in modern medicine.

## AUTHOR CONTRIBUTIONS

**Shwetha B. Nagarajan**, **Sanjeevi Ramakrishnan** and **Anuradha Jayaraman**: Conceptualization (equal); data curation (equal); visualization (equal); writing—original draft (equal); investigation (equal); writing—original draft (equal); project administration (equal); writing—review & editing (equal).

## CONFLICT OF INTEREST STATEMENT

The authors declare no conflict of interest.

## ETHICS STATEMENT

Not applicable.

## INFORMED CONSENT

Not applicable.

## Data Availability

Data sharing is not applicable to this article as no data sets are generated and analysed during the current study.
